# Postoperative respiratory failure necessitating transfer to the intensive care unit in orthopedic surgery patients: risk factors, costs, and outcomes

**DOI:** 10.1186/s13741-016-0044-1

**Published:** 2016-08-02

**Authors:** Roman Melamed, Lori L. Boland, James P. Normington, Rebecca M. Prenevost, Lindsay Y. Hur, Leslie F. Maynard, Molly A. McNaughton, Tyler G. Kinzy, Adnan Masood, Mehdi Dastrange, Joseph A. Huguelet

**Affiliations:** 1 Department of Critical Care Medicine, Abbott Northwestern Hospital, 800 East 28th Street, Minneapolis, MN 55407 USA; 2 Division of Applied Research, Allina Health, 2925 Chicago Ave South, Minneapolis, MN USA; 3 Department of Pharmacy, Abbott Northwestern Hospital, 800 East 28th Street, Minneapolis, MN USA; 4 Chronic Pain Team, Abbott Northwestern Hospital, 800 East 28th Street, Minneapolis, MN USA; 5 Internal Medicine Residency Program, Abbott Northwestern Hospital, 800 East 28th Street, Minneapolis, MN USA

**Keywords:** Respiratory failure, Orthopedic procedure, In-hospital, Critical care medicine, Intensive care unit transfer

## Abstract

**Background:**

Postoperative pulmonary complications in orthopedic surgery patients have been associated with worse clinical outcomes. Identifying patients with respiratory risk factors requiring enhanced monitoring and management modifications is an important part of postoperative care. Patients with unanticipated respiratory decompensation requiring transfer to the intensive care unit (ICU) have not been studied in sufficient detail.

**Methods:**

A retrospective case-control study of elective orthopedic surgery patients (knee, hip, shoulder, or spine, *n* = 51) who developed unanticipated respiratory failure (RF) necessitating transfer to the ICU over a 3-year period was conducted. Controls (*n* = 153) were frequency matched to cases by gender, age, and surgical procedure. Patient and perioperative care factors, clinical outcomes, and cost of care were examined.

**Results:**

Transfer to the ICU occurred within 48 h of surgery in 73 % of the cases, 31 % required non-invasive ventilation, and 18 % required mechanical ventilation. Cases had a higher prevalence of chronic obstructive pulmonary disease (COPD), obstructive sleep apnea (OSA), and regular psychotropic medication use than controls. Cases received more intravenous opioids during the first 24 postoperative hours, were hospitalized 4 days longer, had higher in-hospital mortality, and had excess hospitalization costs of US$26,571. COPD, OSA, preoperative psychotropic medications, and anesthesia time were associated with risk of RF in a multivariate analysis.

**Conclusions:**

Unanticipated RF after orthopedic surgery is associated with extended hospitalization, increased mortality, and higher cost of care. Hospital protocols that include risk factor assessment, enhanced monitoring, and a cautious approach to opioid use in high-risk patients may reduce the frequency of this complication.

## Background

Demand for elective orthopedic procedures in the USA is expected to grow (Kurtz et al. 2007), and these procedures can be associated with serious cardiorespiratory complications (Dahl 1997). Identifying patients at high risk for postoperative pulmonary complications (PPC) and managing modifiable risk factors is essential, but effective strategies are limited (Bapoje et al. 2007). PPC develop in 7–14 % of patients undergoing spine or major joint surgery (Imposti et al. 2010; Lee et al. 2011; Lee et al. 2012; Lee et al. 2013; Ryu et al. 2010), and their occurrence is associated with longer length of stay and higher mortality (Lee et al. 2011; Lee et al. 2013; Lawrence et al. 2002; Liu et al. 2011; Smith et al. 2012). Potential PPC include pneumonia, atelectasis, pleural effusion, aspiration, airway compromise, acute respiratory distress syndrome, transfusion-related acute lung injury, fat or pulmonary embolism, opiate-related respiratory depression, and respiratory arrest (Imposti et al. 2010; Lee et al. 2012; Issack et al. 2009; Raw et al. 2003; Taylor and Gropper 2006), and their occurrence may be affected by the surgical site (Miura et al. 1996; Suk et al. 2006; Yoshida et al. 2007; Sagi et al. 2002).

Risk factors for PPC include advanced age, higher American Society of Anesthesiologists (ASA) class, smoking, chronic obstructive pulmonary disease (COPD), congestive heart failure (CHF), chronic renal failure, diabetes, and obstructive sleep apnea (OSA) (Imposti et al. 2010; Lee et al. 2012; Lee et al. 2013; Liu et al. 2011; Fu et al. 2011; Gupta et al. 2001; Memtsoudis et al. 2011; Mokhlesi et al. 2013; Moller et al. 2003; Roche et al. 2005; Weis et al. 1997). Intraoperative risk factors include surgical invasiveness, longer anesthesia time, thoracic-level procedure, and increased blood loss (Imposti et al. 2010; Lee et al. 2012; Sagi et al. 2002). The use of opioids in the postoperative period is also of concern as it may suppress central respiratory drive and cough and induce a monotonous respiratory pattern (Taylor and Gropper 2006), particularly in the elderly (Egbert 1996; Petre et al. 2012). However, studies of PPC have largely focused on preoperative and intraoperative predictors, and only one has quantified postoperative opioids in orthopedic surgery patients (Petre et al. 2012).

Estimated additional total costs related to PPC range from US$5983 to US$120,579 per procedure, with higher costs associated with mechanical ventilation or tracheostomy (Sabate et al. 2014). Opioid-related PPC are associated with increased lengths of stay and costs and are associated with higher opioid doses (Oderda et al. 2007).

We conducted a case-control study of orthopedic surgery patients who were transferred from the post-anesthesia care unit (PACU) to the general orthopedic floor but subsequently developed respiratory failure (RF) requiring transfer to the intensive care unit (ICU). The study objectives were to describe the causes of RF, to investigate potential perioperative factors which can cause postoperative deterioration, and to examine outcomes such as mortality and total cost of care in cases and controls.

## Methods

We conducted a retrospective frequency-matched case-control study (1:3) among adult patients who underwent major orthopedic surgery between December 1, 2010, and November 30, 2013, at Abbott Northwestern Hospital, a tertiary hospital in Minneapolis, MN, that performs approximately 4000 spine and 2000 major joint surgeries annually. The study protocol was approved by the Schulman Institutional Review Board with a waiver of informed consent.

Cases were patients who underwent elective or semi-elective spine or major joint surgery and were postoperatively initially stable (i.e., oxygen saturation >90 % while receiving supplemental oxygen via nasal cannula and not requiring respiratory or hemodynamic support beyond standard care) but then experienced unanticipated RF and were subsequently transferred to the ICU. We queried the hospital’s electronic health record (EHR) for procedural codes representing specific spine or major joint surgery based on the International Classification of Diseases 9th Revision (ICD-9). For descriptive purposes, patients were grouped by anatomical site of the surgery, i.e., knee, hip, spine (cervical), spine (non-cervical), and shoulder. We then restricted this list of patients to those who were initially stable and transferred directly from the PACU to a medical/surgical unit. Instances of unanticipated RF were then identified using a two-step process. First, we identified patients who were transferred to the ICU and had any of the following ICD-9 discharge diagnosis codes: 518.81 (acute RF), 518.84 (acute and chronic RF), 518.51 (acute RF following trauma/surgery), 518.52 (other pulmonary insufficiency following trauma/surgery), 518.82 (acute pulmonary insufficiency), or 786.09 (respiratory distress). The medical records of these patients were then reviewed independently by two physicians (RM and MD) who confirmed RF if any of the following criteria were met: pO_2_ <60 mmHg on room air or pCO_2_ >45 mmHg; increase in pCO_2_ from baseline or increase in supplemental oxygen requirements; or documentation of the patient’s inability to protect airway or maintain adequate gas exchange. Controls were selected from among patients who underwent the same surgical procedures as the cases during the study timeframe but who were neither transferred to the ICU nor developed RF. Three controls were randomly selected for each case, frequency-matched on surgical procedure, gender, and 10-year age category. Patients <18 years of age or who did not provide consent for use of their EHR data for research were excluded.

Patient demographics and event details including preoperative ASA class, length of stay, and in-hospital mortality were extracted from the EHR. ICU length of stay and ventilatory support details were extracted for cases. Two physicians (RM and MD) independently reviewed patient care records to determine the primary cause of RF; conditions present on admission such as COPD, OSA, and regular use of psychotropic medications (defined as antipsychotics, antidepressants, benzodiazepines, sleep aides, or muscle relaxants); and total operative anesthesia time (minutes). Total cost of care was obtained via financial databases maintained by the hospital.

Clinicians with expertise in pain management (LYH, LFM, MAM) reviewed EHR data and quantified postoperative opioid use. Use of patient-controlled analgesia (PCA) was categorized as none, incremental dosing only, or a combination of a continuous basal rate and incremental dosing. Total morphine equivalents (milligrams) were computed for 12 consecutive 8-h intervals from the end of surgery up to 96 h after surgery or patient discharge (whichever occurred sooner) using a standardized conversion tool developed by Allina Health Pharmacy Services based on established methods (McPherson 2009). Postoperative use of other sedatives such as diazepam and lorazepam was also documented.

### Analysis

Continuous variables were described using means or medians based on results of Shapiro-Wilk tests for normality, and values in cases and controls were compared using *t* tests or Wilcoxon-Mann-Whitney rank-sum tests accordingly. Categorical data were described using counts and proportions, with differences between cases and controls assessed using Fisher’s exact test. Unconditional logistic regression models were used to estimate adjusted odds ratios and 95 % confidence intervals for associations between hypothesized risk factors and RF. All analyses were conducted using Stata 12.1 (StataCorp LP, College Station, TX).

## Results

Fifty-one cases of unanticipated postoperative RF occurred among 14,465 qualifying surgical procedures performed during the study timeframe (0.4 %). The most common procedure-related diagnoses were spinal stenosis and degenerative joint disease. There was no significant difference in obesity between cases and controls, but cases had a statistically significantly higher prevalence of COPD, OSA, chronic kidney disease (CKD), and psychotropic medication use and higher ASA class (Table 1). Among the 35 patients with diagnosed OSA, 35 % of cases (5/18) and 71 % of controls (*n* = 12/17) were using continuous positive airway pressure (CPAP) at home at the time of admission (*p* = 0.02), and among 16 patients with COPD, 18 % of cases (2/11) and 20 % of controls (1/5) were using home oxygen.

**Table 1:** Surgical procedures, patient characteristics, and conditions present on admission

Variable	Cases (*n* = 51)	Controls (*n* = 153)	*p* value*
Surgical procedure type			
Knee	37 % (19)	37 % (57)	
Spine (cervical)	41 % (21)	41 % (63)	
Spine (non-cervical)	16 % (8)	16 % (24)	
Hip	4 % (2)	4 % (6)	
Shoulder	2 % (1)	2 % (3)	
Age, years	66 (34–88)	67 (32–90)	
Age category, years			
31–40	2 % (1)	2 % (3)	
41–50	6 % (3)	6 % (9)	
51–60	22 % (11)	22 % (33)	
61–70	33 % (17)	33 % (51)	
71–80	22 % (11)	22 % (33)	
81+	16 % (8)	16 % (24)	
Male	35 % (18)	35 % (54)	
Body mass index (BMI), kg/m^2^			
<25	14 % (7)	17 % (26)	0.67
25.0–29.9	43 % (22)	37 % (56)	0.41
30.0–35.0	18 % (9)	25 % (38)	0.34
>35.0	26 % (13)	22 % (33)	0.57
ASA class			
1	0 % (0)	2 % (3)	0.58
2	41 % (21)	69 % (105)	<0.001
3	57 % (29)	29 % (44)	<0.001
4	2 % (1)	1 % (1)	0.44
Conditions present on admission			
Asthma	20 % (10)	10 % (16)	0.10
COPD	22 % (11)	3 % (5)	<0.001
OSA	35 % (18)	11 % (17)	<0.001
Obesity (BMI ≥ 30 kg/m^2^)	43 % (22)	46 % (71)	0.75
CHF	10 % (5)	2 % (3)	0.03
CKD	22 % (11)	6 % (9)	0.002
Restrictive lung disease	4 % (2)	1 % (1)	0.16
Opioid use	41 % (21)	34 % (52)	0.40
Smoking history	57 % (29)	37 % (57)	0.01
Regular use of psychotropic medication	71 % (36)	37 % (56)	<0.001
Diabetes	18 % (9)	11 % (17)	0.23

Results are expressed as mean (range) or percent (*n*) unless otherwise indicated

*ASA* American Society of Anesthesiologists, *BMI* body mass index, *COPD* chronic obstructive pulmonary disease, *OSA* obstructive sleep apnea, *CHF* congestive heart failure, *CKD* chronic kidney disease

*Fisher’s exact test; *p* values not shown for matched variables

The most common primary causes of RF were decreased levels of consciousness with depressed respiratory drive (with or without laboratory confirmation of hypercarbia), pneumonia, CHF, and COPD exacerbation (Table 2). There were three cases of cardiac arrest: two related to respiratory depression and one due to acute myocardial infarction. Most cases (72 %) developed RF within the first 2 postoperative days, with a median time from the end of surgery to the ICU transfer of 42 h.

**Table 2:** Cause of respiratory failure and timing of ICU transfer in cases

Variable	Cases (*n* = 51)
Cause of respiratory failure	
Altered LOC with respiratory depression	20 % (10)
Hypercapnic respiratory failure	18 % (9)
Pneumonia	16 % (8)
CHF	10 % (5)
COPD exacerbation	8 % (4)
Aspiration	6 % (3)
Cardiac arrest	6 % (3)
Sepsis	6 % (3)
Pulmonary embolism	4 % (2)
Upper airway edema/obstruction	4 % (2)
Fat embolism	2 % (1)
OSA/OHS	2 % (1)
Total	100 % (51)
Day transferred to ICU	
Day 0	12 % (6)
Day 1	28 % (14)
Day 2	33 % (17)
Days 3–4	20 % (10)
Days 5–6	8 % (4)
Time between end of surgery and transfer to ICU, hours	42 (4–107)

Results are expressed as mean (range) or percent (*n*) unless otherwise indicated

*ICU* intensive care unit, *LOC* loss of consciousness, *CHF* congestive heart failure, *COPD* chronic obstructive pulmonary disease, *MI* myocardial infarction, *OSA* obstructive sleep apnea, *OHS* obesity hypoventilation syndrome

Spine and shoulder surgery patients received general anesthesia while regional anesthesia was used for knee and hip procedures. Cases generally had longer anesthesia times and higher estimated blood loss than controls, but there was no significant difference between the groups with regard to transfusion requirements (data not shown). Among spine surgery patients, cases received slightly higher doses of intraoperative opioids, but this difference was not observed in major joint surgery patients (Table 3).

**Table 3:** Intraoperative and postoperative opioid use, by spine and major joint procedures

	Spine surgery			Major joint surgery		
Variable	Cases	Controls	*p* value*	Cases	Controls	*p* value*
	*n* = 29	*n* = 87		*n* = 22	*n* = 66	
Total intraoperative opioids (ME), mg	115 (95–185)	85 (69–120)	0.006	18 (15–45)	23 (15–68)	0.77
Postoperative PCA						
Incremental only	21 % (6)	15 % (13)	0.56	0 % (0)	2 % (1)	1.00
Continuous and incremental	69 % (20)	36 % (31)	0.002	0 % (0)	3 % (2)	1.00
None	10 % (3)	49 % (106)	<0.001	100 % (22)	95 % (63)	0.57
Total postoperative opioids (ME), mg						
First 8 h	40 (0–470)	33 (0–179)	0.42	26 (0–107)	9 (0–240)	0.01
First 24 h	117 (10–1414)	87 (0–448)	0.05	109 (15–224)	64 (0–499)	0.03
First 48 h	240 (10–981)	153 (0–857)	0.37	130 (65–397)	137 (0–934)	0.97
First 72 h	416 (20–1451)	242 (15–1262)	0.33	174 (112–236)	190 (17–1480)	0.79
First 96 h	716 (408–1023)	331 (37–1667)	0.64	112 (112–112)	189 (22–2110)	0.59
Use of other sedative during postoperative course	83 % (24)	48 % (42)	0.001	64 % (14)	52 % (34)	0.46

Results are expressed as median (range) or percent (*n*) unless otherwise indicated

*PCA* patient-controlled analgesia, *ME* morphine equivalents

*Fisher’s exact test or Wilcoxon rank-sum test as appropriate

Overall, cases received more intravenous opioids during the first 24 postoperative hours than controls (median 110 vs 73 mg, *p* = 0.006), and opioid use was examined separately for patients with spine versus major joint procedures (Table 3). In spine surgery patients, the use of any PCA was more frequent in cases than in controls, and cases were more likely to have used a combination of continuous and incremental PCA than controls. Total postoperative opioids were generally higher in spine surgery patients than in major joint surgery patients. In major joint surgery patients, doses received in the first 8- and 24-h intervals were positively associated with the development of RF, with the median total dose in the first 8-h interval nearly three times higher in cases than in controls. Data in spine surgery patients suggest a similar pattern of higher opioid doses in cases across the 96-h postoperative interval. Use of additional sedatives during the postoperative course was associated with RF in spine surgery patients.

Among the cases and controls with previously diagnosed OSA, CPAP was started in the PACU in 11 % of cases (2/18) and 29 % of controls (5/17; *p* = 0.23). Non-invasive ventilation (NIV) was ultimately initiated in 56 % of cases who had OSA (10/18), commonly as a rescue therapy after the development of respiratory insufficiency.

Nine cases required mechanical ventilation (median hours = 56), 16 were managed with non-invasive ventilation, and in-hospital mortality was 6 % in cases and 0 % in controls (*p* = 0.003, Table 4). Average hospitalization cost was significantly higher in cases (US$46,456) than in controls ($19,885, *p* < 0.001).

**Table 4:** Hospitalization details and outcomes

Variable	Cases (*n* = 51)	Controls (*n* = 153)	*p* value*
Mechanical ventilation	18 % (9)		
Non-invasive ventilatory support (BiPAP)	31 % (16)		
Total ventilation time, h	56 (1–347)		
ICU length of stay, days	1.6 (0.2–17)		
Hospital length of stay, days	7 (2–41)	3 (1–8)	<0.001
In-hospital mortality	6 % (3)	0 % (0)	0.003
Cost of hospitalization, $US^a^	46,456	19,885	<0.001
Discharge destination			
Died	6 % (3)	0 % (0)	0.003
Home	55 % (28)	70 % (107)	0.046
SNF/rehabilitation	39 % (20)	30 % (46)	0.193

Results are expressed as median (range) or percent (*n*) unless otherwise indicated

*ICU* intensive care unit, *SNF* skilled nursing facility

*Wilcoxon rank-sum test, two-sample *t* test, or Fisher’s exact test, as appropriate

^a^Mean US dollars

Multivariate logistic regression models initially included the three matching variables and the following covariates that were statistically significant in univariate analyses (*p* ≤ 0.01): ASA class, COPD, OSA, CKD, smoking, psychotropic medication use, total operative anesthesia time, estimated blood loss, and opioids in the first 24 postoperative hours. Due to collinearity with comorbidities, ASA class was omitted from the final model, the results of which are presented in Table 5. COPD, OSA, CKD, psychotropic medication use, and total anesthesia time were all independently associated with risk of RF, but opioids in the first 24 postoperative hours were not associated with RF in the presence of these other factors.

**Table 5:** Adjusted odds ratios and 95 % confidence intervals of unplanned postoperative respiratory failure according to selected risk factors

Variable	OR	95 % CI
COPD	4.87	(1.24–19.21)
OSA	4.79	(1.81–12.67)
CKD	4.83	(1.36–17.17)
Regular use of psychotropic medication	4.02	(1.58–10.26)
Total operative anesthesia time	1.27	(1.10–1.48)
Total opioid dose in the first 24-h postoperative interval	1.02	(0.98–1.06)

Results from a logistic regression model that included gender, surgical procedure type, age (10-year categories), and all variables shown in the table

*OR* odds ratio, *CI* confidence interval, *COPD* chronic obstructive pulmonary disease, *OSA* obstructive sleep apnea, *CKD* chronic kidney disease

## Discussion

Previous studies of PPC have been conducted in more heterogeneous groups of surgical patients and have not distinguished between patients who were initially stable versus those who were directly transferred to the ICU after surgery. To our knowledge, this is the first case-control study of postoperative RF in orthopedic surgery patients to examine detailed information on postoperative opiate administration and to provide information on the primary causes of RF, clinical outcomes, and cost of care.

Postoperative RF is a significant complication in terms of increased mortality, length of stay, and cost (Dimick et al. 2004; Khuri et al. 2005), so there is strong interest in developing and validating tools that accurately identify high-risk patients for targeted intervention (Sabate et al. 2014; Canet and Gallart 2013). A scorecard quantifying the risk of opioid-related respiratory depression (Katie Felhofer 2013) and two scoring algorithms designed specifically for predicting postoperative RF have been proposed (Arozullah et al. 2000; Gupta et al. 2011). The demographic homogeneity of the surgical population that yielded one approach has been cited as a serious limitation (Arozullah et al. 2000), and while the score developed recently by Gupta et al. (Gupta et al. 2011) appears promising in terms of more generalizable use, only 4 % of the patients underwent orthopedic procedures and their criteria for RF were stricter (i.e., patients requiring intubation or mechanical ventilation for >48 h). In addition, only one of the five criteria used in this score (i.e., ASA class) would have any significant variability in the patients studied here.

Consistent with previous studies, we found that risk of RF was associated with preexisting COPD, OSA, CHF, and CKD, as well as with smoking, longer anesthesia time, and higher intraoperative blood loss (Imposti et al. 2010; Lee et al. 2011; Lee et al. 2012; Sagi et al. 2002; Gupta et al. 2001; Moller et al. 2003; Roche et al. 2005). Advanced age was a strong risk factor for PPC in previous studies (Imposti et al. 2010; Lee et al. 2012; Canet and Gallart 2013; Smetana et al. 2006), although we find it noteworthy that almost 30 % of our cases were under the age of 60.

OSA is a common disorder associated with increased morbidity and mortality (Peppard et al. 2013; Young et al. 2009). In a large analysis of orthopedic surgery patients, OSA was present in 8.4 % and was associated with increased pulmonary and cardiac complications, use of intensive care, ventilatory support, and longer hospitalization (Memtsoudis et al. 2014). The postoperative state has been shown to exacerbate OSA symptoms and even cause sleep-disordered breathing in patients without OSA (Chung et al. 2014). In our study, cases with OSA had lower rates of compliance with home CPAP than controls. A validated OSA screening tool as described by Chung et al (Chung et al. 2016) could potentially be used to prompt earlier NIV use and enhanced postoperative monitoring in orthopedic surgical patients with diagnosed or suspected OSA.

Postoperative pain management with opioids presents significant challenges in orthopedic surgery patients (Taylor and Gropper 2006). Opioid-induced oversedation and associated respiratory depression is a principal concern, particularly in the presence of OSA, obesity, advanced age, preexisting pulmonary or cardiac disease, and longer anesthesia times (The Joint Commission 2012). But while opioid use may be an important risk factor for PPC, it is noticeably absent from the summarized evidence presented in two recent systematic reviews (Sabate et al. 2014; Canet and Gallart 2013). Data from the only other study that has quantified postoperative opioids when examining PPC in orthopedic surgery patients (Petre et al. 2012) suggest that higher doses of opioids may be associated with increased risk of PPC. In our study, cases had received significantly more opioids than controls in the first 8–24 postoperative hours and typically required transfer to the ICU within the first 48 h after surgery. The initial 24–48 postoperative hours thus may represent a particularly sensitive period when patients are prone to the development of respiratory insufficiency and the side effects of opioids, a theory that is supported by evidence that postoperative patients who experience analgesic-related respiratory events are more likely to have those events in the first 24 h after surgery (Taylor et al. 2005). Our finding that the use of additional sedatives during the postoperative course was also associated with RF further supports this hypothesis, but this topic requires further study.

Cases in our study remained hospitalized an average of 4 days longer than controls, and on average, total costs were US$26,571 more for their hospitalization. Nearly one in five cases in our study required mechanical ventilation, and previous studies estimate that patients requiring intubation have longer hospital stays and higher total charges (Liu et al. 2011; Gupta et al. 2001; Mokhlesi et al. 2013).

Our results highlight the need for postoperative protocols that incorporate the type and complexity of the surgical procedure, perioperative events, individual patient risk factors, and facility-specific workflow and resources. Future studies should evaluate the effect on patient outcomes of clinical pathways that include patient assessment in the immediate postoperative period and a standardized hand-off process from anesthesia to the medical team. A mechanism for alerting the entire multidisciplinary team (e.g., nursing, hospitalists, respiratory care, pharmacists, surgeons) to the patient’s risk factors and pain control, respiratory support, and monitoring needs would be an essential component of such protocols. This approach may reduce the frequency of both severe cases of RF requiring ICU transfer and respiratory events of lesser severity that can be managed outside the ICU.

Limitations of this study include the fact that we did not ascertain the entire spectrum of PPC in elective orthopedic surgery patients at our facility during the study timeframe. Second, given the single-center study design and narrowly defined patient population, our results may not be applicable to other treatment centers or surgical populations. Third, a detailed evaluation of the patient’s preoperative cardiorespiratory and nutritional status was limited by the retrospective study design. Fourth, we were unable to blind the clinicians who computed total opioids from the case status of patients, but we do not believe this resulted in systematic differences in the assessment given the objective nature of the computation.

## Conclusions

Unanticipated RF requiring ICU transfer occurs in less than 1 % of patients who undergo elective orthopedic surgery, but despite their rarity, these events are associated with higher mortality, longer length of stay, and significantly increased hospital costs. Hospitals, and particularly high-volume orthopedic surgery centers, should consider a structured multidisciplinary team approach to postoperative management that involves systematic risk factor assessment, enhanced monitoring techniques, a judicious approach to pain management, and early identification and treatment of PPC.

## Abbreviations

ASA class, American Society of Anesthesiologists physical status classification (system for assessing the fitness of patients before surgery); CHF, congestive heart failure; CKD, chronic kidney disease; COPD, chronic obstructive pulmonary disease; EHR, electronic health record; ICU, intensive care unit; LFM, Leslie F. Maynard, CNP (author); LYH, Lindsay Y. Hur, PharmD (author); MAM, Molly A. McNaughton, CNP (author); MD, Mehdi Dastrange, MD (author); OSA, obstructive sleep apnea; PACU, post-anesthesia care unit; PCA, patient-controlled analgesia; PPC, postoperative pulmonary complications; RF, respiratory failure; RM, Roman Melamed, MD (author)
